# Disease characteristics, prognosis and miglustat treatment effects on disease progression in patients with Niemann-Pick disease Type C: an international, multicenter, retrospective chart review

**DOI:** 10.1186/s13023-019-0996-6

**Published:** 2019-02-07

**Authors:** Mercedes Pineda, Katarína Juríčková, Parvaneh Karimzadeh, Miriam Kolnikova, Vera Malinova, Jose Luis Insua, Christian Velten, Stefan A. Kolb

**Affiliations:** 1 Hospital Sant Joan de Deu, Passeig de Sant Joan de Deu, 2, Esplugues de Llobregat, 08950 Barcelona, Spain; 2 Department of Pediatrics, Centre for Inborn Errors of Metabolism, Comenius University Children’s Hospital, Bratislava, Slovakia; 3 Department of Paediatric Neurology, Paediatric Neurology Research Centre, Shahid Beheshti University of Medical Sciences, Mofid Children Hospital, Tehran, Iran; 4 Department of Child Neurology, Comenius University Medical School and National Institute of Children’s Diseases, Bratislava, Slovakia; 5 Department of Pediatrics and Adolescent Medicine, First Faculty of Medicine, Charles University and General University Hospital, Prague, Czech Republic; 6 Syntax for Science SL, Mallorca, Spain; 7 Actelion Pharmaceuticals Ltd., Allschwil, Switzerland

**Keywords:** Niemann-pick disease type C, NP-C, Disease characteristics, Prognosis, NP-C disability scales

## Abstract

**Background:**

Niemann-Pick disease Type C (NP-C) is a lysosomal lipid storage disorder characterized by progressive neurodegenerative symptomatology. The signs and symptoms of NP-C vary with age at disease onset, and available therapies are directed at alleviating symptoms and stabilizing disease progression. We report the characteristics and factors related to disease progression, and analyze the effect of miglustat treatment on disease progression and patient survival using NP-C disability scales.

**Methods:**

This retrospective, observational chart review included patients with NP-C from five expert NP-C centers. Patient disability scores were recorded using three published NP-C disability scales, and a unified disability scale was developed to allow comparison of data from each scale. Disease progression was represented by scores on the unified NP-C disability scale. Patients were stratified as infantile (< 4 years), juvenile (≥ 4 − < 16 years), and adult (≥ 16 years) based on age at diagnosis, and treated ≥1 year and non-treated/treated < 1 year based on the duration of miglustat treatment.

**Results:**

The analysis included 63 patients; the majority (61.9%) were on miglustat therapy for ≥1 year. Ataxia and clumsiness/frequent fall were the most common neurologic symptoms across age groups, whereas, hypotonia and delayed developmental milestones were specific to infantile patients. In both infantile and juvenile patients, visceral signs preceded diagnosis and neurologic signs were noted at or shortly after diagnosis. Adult patients presented with a range of visceral, neurologic, and psychiatric signs in years preceding diagnosis. Patients on miglustat therapy for ≥1 year had a lower mean annual disease progression compared with those untreated/treated < 1 year (1.32 vs 3.54 points/year). A significant reduction in annual disease progression in infantile patients, and a trend towards reduced disease progression in juvenile patients after ≥1 year of miglustat treatment, translated into higher age at last contact or death in these groups.

**Conclusions:**

The type and onset of symptoms varied across age groups and were consistent with descriptions of NP-C within the literature. Miglustat treatment was associated with a reduced rate of disability score worsening in infantile and juvenile patients, both in agreement with increased age at last contact.

**Electronic supplementary material:**

The online version of this article (10.1186/s13023-019-0996-6) contains supplementary material, which is available to authorized users.

## Background

Niemann-Pick disease Type C (NP-C) is a rare, fatal, autosomal recessive, lysosomal lipid storage disease characterized by progressive neurodegenerative symptomatology [1–3]. The incidence of NP-C is estimated to range between 1:100,000 and 1:150,000 live births or to be as low as 1:89,229 conceptions [3–5] and is caused by mutations in either the *NPC1* gene, which accounts for 95% of cases, or the *NPC2* gene [1, 6, 7].

The age of onset of NP-C can range from the perinatal period to adulthood, with symptomatology varying with age of onset. Early-onset NP-C tends to be more severe and rapidly progressive than adult-onset NP-C [3, 7]. Patients who develop NP-C during early infancy often present with visceral manifestations such as splenomegaly, hepatomegaly, and cholestasis, with varying degrees of neurologic signs and symptoms including delayed developmental milestones, hypotonia, and dystonia [8, 9]. In adolescence or adulthood, patients tend to present with varying combinations of progressive neurologic deficits such as ataxia, dystonia, and/or dementia or major psychiatric illness [9]. Vertical supranuclear saccade palsy/vertical supranuclear gaze palsy (VSGP) is the most common neurologic symptom, and is often overlooked during the initial differential diagnosis [3].

The diagnosis of NP-C is often challenging, owing to the heterogeneous and nonspecific clinical presentation of patients with NP-C. This may result in substantial diagnostic delays, with a mean delay of 4.1 years from onset of neurologic symptoms reported [1, 10, 11]. Currently there is no cure for NP-C, and the therapies are directed to alleviate symptoms of the disease [11]. Miglustat (Zavesca®, Actelion Pharmaceuticals Ltd.) is currently the only therapy available to treat the neurologic manifestations of NP-C and is approved in several countries in Asia, Europe, Middle East, North America, Oceania, and South America. Miglustat has been shown to delay or stabilize the disease course of NP-C [11–14]. Several observational studies have utilized disease-specific disability scales for patients with NP-C. These scales offer objective and semi-quantitative assessment of disease progression and responses to therapy, and provide additional evidence for the benefits of miglustat treatment [13–17].

Here we report data from an observational, retrospective chart review conducted to identify characteristics and factors related to disease progression and assess the effect of miglustat treatment on the rate of disease progression and patient survival using NP-C disability scales [14, 15, 17].

## Methods

### Study design and population

This was a retrospective, international, multicenter, observational chart review of data collected between February 2016 and December 2016 from five expert NP-C centers providing miglustat treatment, in the Czech Republic, Iran, Slovakia (two centers), and Spain. The participating site or physician was responsible for obtaining ethical approval. Informed consent was obtained from either the patient or their parents/legal guardians, according to local laws. Eligible patients had a confirmed diagnosis of NP-C by either classical filipin staining with or without two known pathogenic *NPC* mutations; variant filipin staining with two confirmed known pathogenic *NPC* mutations; or two confirmed known pathogenic *NPC* mutations. Patients with lysosomal storage diseases or enzyme deficiency diseases other than NP-C, and a variant filipin staining without confirmatory genetic diagnosis of NP-C by two confirmed known *NPC* mutations, were excluded from the study.

### Data collection

In this retrospective chart review, patient identification was anonymized for all persons involved in analysis and review of the data, including the participating investigators and the sponsor. Data were collected using a web-based electronic data capture (EDC) system, which was exported into SAS® (SAS Institute Inc., Cary, NC) for analysis. Data collection included demographics, disability scale and scores, medical history, NP-C diagnosis, onset date of signs and symptoms, and treatment history including miglustat treatment exposure. If necessary, and in agreement with the investigators, additional data were retrieved retrospectively from patient records.

### Objectives and assessments

Our primary objective was to analyze disease progression, represented by scores on a unified NP-C disability scale (or appropriately mapped scores), in patients diagnosed with NP-C, to assess the effect of miglustat treatment over time for different clinical forms of NP-C defined by age of diagnosis. Other objectives were to identify characteristics and factors related to disease progression and survival, and conduct a retrospective comparison of miglustat treatment with pre-miglustat, where data were available.

Patient disability scores were recorded using one of 3 NP-C disability scales: the Iturriaga et al. 4-domain NP-C disability scale [15], the Pineda et al. 6-domain NP-C disability scale [14], and the Fecarotta et al. 6-domain NP-C severity rating scale [17]. For simplicity, we refer to each scale by its first named author. In order to allow comparison of data from each scale, a unified NP-C disability scale was produced for mapping domains and scores from the Iturriaga, Pineda, and Fecarotta scales (Additional file 1: Table S1). Harmonization of data was in concordance with authors and best clinical practice. The unified NP-C disability scale consists of the following 6-domains: Ambulation, Manipulation, Language, Swallowing, Seizures, and Ocular movements. The unique domains (Dystonia, Developmental delay/cognitive impairment) from either the Iturriaga or the Fecarotta scale were omitted. A score of 1 on the Iturriaga scale and not applicable (n/a) on the Pineda scale were recorded as ‘0’ (normal, absence of abnormalities) on the unified scale. Two domains (Seizures and Ocular movements) that are part of the unified scale but not included in the Iturriaga scale were imputed from patient medical records using the Last Observation Carried Forward method and coherence rules. Imputation was also used to create additional timepoints for patients who had only one recorded visit where a disability scale had been used. Additional timepoints were imputed from medical records into disability scale scores, allowing disease progression to be monitored. A conservative approach to imputing symptoms (using the lowest quantitative score in the 6-domain disability scale) was selected to prevent overestimating the scores (Additional file 1: Table S2).

Disease progression (disability scale increase/year) was assessed using quantitative statistics. Patients were stratified by age at diagnosis according to previously published age categories: infantile (< 4 years), juvenile (≥ 4 − < 16 years), and adult (≥ 16 years) [8, 9]. Patients were also categorized based on the duration (defined by the sum of all) of miglustat treatment into non-treated and treated for < 1 year (herein referred to as *control*) and treated for ≥1 year (herein referred to as *treated*). One year was selected as the cut-off for the control cohort, as it may take up to 1 year to see a treatment effect following initiation of miglustat therapy [7, 18]. Two timepoints were defined. The *baseline visit* was defined as the first assessment post-diagnosis in both the control and treated groups. The *last visit* was defined as the last assessment post-diagnosis in the control group and the last assessment post-diagnosis and post-treatment initiation in the treated group.

### Data analyses

The analysis population comprised all patients included in the database. Numerical variables in demographics and baseline characteristics were based on descriptive statistics. Annual progression was defined as the change from baseline to last visit divided by the number of years. Annual progression, absolute values, and changes from baseline values were analyzed by descriptive statistics. For patient disposition, descriptive statistics for first event, diagnosis, miglustat treatment initiation, last contact, and death were reported. First event was defined as the date of first sign or symptom (either neurologic, visceral, or psychiatric) included in the unified disability scale. Population survival was calculated as time from date of diagnosis to last contact or death. A linear regression model was used to assess the effect of miglustat treatment on the disease progression rate. The relationship between disability scores and time (time from the first sign or symptom) was determined, and the resulting linear equation estimates, standard error, 95% confidence interval (CI), and *p*-values were derived.

## Results

### Patients and disease characteristics

A total of 63 patients were included in this retrospective analysis. Based on age at diagnosis, patients were categorized into infantile (*n* = 18), juvenile (*n* = 22), and adult (*n* = 23) groups. The mean (standard deviation [SD]) time between first neurologic symptoms and miglustat therapy was 4.71 (6.05) years; mean (SD) time between diagnosis and miglustat treatment was 1.91 (3.19) years. The majority of patients (82.5%) received miglustat treatment, and 61.9% of patients received miglustat for more than 1 year (Table 1). The median (range) duration of miglustat treatment was 2.89 (0.01–9.7) years overall and 0.16 (0.01–1.0) years for patients who received miglustat treatment for < 1 year.

**Table 1:** Baseline characteristics and treatment history of patients with NP-C

Characteristics	Diagnosis			
	Infantile	Juvenile	Adult	Total
	(*n* = 18)	(*n* = 22)	(*n* = 23)	(*n* = 63)
Gender, n (%)				
Male	12 (66.7)	14 (63.6)	11 (47.8)	37 (58.7)
Female	6 (33.3)	8 (36.4)	12 (52.2)	26 (41.3)
Family history, n (%)				
Parent or sibling with NP-C	2 (11.1)	4 (18.2)	9 (39.1)	15 (23.8)
Cousin with NP-C	1 (5.6)	1 (4.5)	1 (4.3)	3 (4.8)
Other relatives with NP-C	0 (0.0)	1 (4.5)	0 (0.0)	1 (1.6)
Presence of *NPC1* mutation, n (%)	18 (100)*	21 (95.5)	23 (100.0)	62 (98.4)
Presence of *NPC2* mutation, n (%)	1 (5.6)*	1 (4.5)	0 (0.0)	2 (3.2)
Age at diagnosis, mean (SD), years	2.15 (1.10)	10.97 (3.82)	25.98 (8.15)	13.93 (11.23)
Treated with miglustat, n (%)	16 (88.9)	18 (81.8)	18 (78.3)	52 (82.5)
Age at miglustat treatment initiation, mean (SD), years	3.62 (4.04)	12.95 (5.28)	24.77 (5.77)	14.17 (10.01)
First neurologic symptom age, years^a^				
n	16	22	23	61
Mean (SD)	2.28 (4.30)	9.46 (4.43)	17.81 (8.69)	10.72 (8.83)
Time between onset of first neurologic symptom and miglustat treatment, years^a^				
n	14	18	18	50
Mean (SD)	1.26 (1.18)	3.35 (4.47)	8.76 (7.44)	4.71 (6.05)
Time from diagnosis to miglustat treatment initiation, years				
n	16	18	18	52
Mean (SD)	1.64 (3.96)	2.14 (3.20)	1.91 (2.51)	1.91 (3.19)
Miglustat total treatment duration, years				
n	16	18	18	52
Mean (SD)	2.63 (1.96)	3.47 (3.25)	4.20 (2.83)	3.46 (2.78)
Miglustat treatment, n (%)				
≥ 1 year	12 (66.7)	13 (59.1)	14 (60.9)	39 (61.9)
< 1 year	4 (22.2)	5 (22.7)	4 (17.4)	13 (20.6)
Non-treated	2 (11.1)	4 (18.2)	5 (21.7)	11 (17.5)
Median age at which 50% of the population showed symptoms, median, years				
Neurologic	1.3	9.8	16.3	
Visceral	0.7	13.8	27.0	
Psychiatric	18.0	10.3	19.8	

*One patient had both *NPC1* and *NPC2* mutations

^a^Includes patients with diagnosis not related to neurologic symptoms

*NP-C* Niemann-Pick disease type C, *SD* standard deviation

**Fig. 1:**
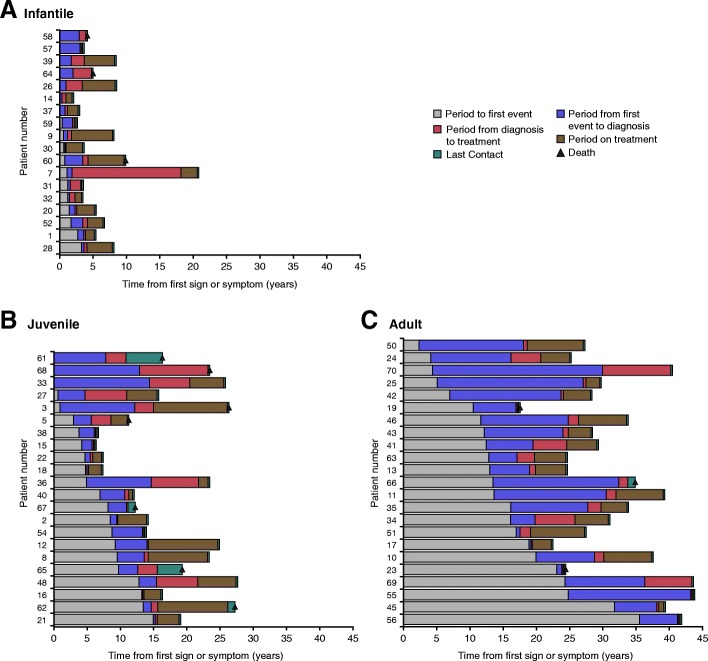
Qualitative description of first event*, diagnosis, miglustat initiation, last contact, and death in (**a**) Infantile, (**b**) Juvenile and (**c**) Adult age groups. *First event was defined as the first sign or symptom that is included in the 6-domain unified disability scale. From the EDC medical history, first sign or symptom could be either neurologic, visceral, or psychiatric. EDC, electronic data capture

There was a lag between the mean (SD) age at neurologic onset (10.72 [8.83] years) and age at diagnosis (13.93 [11.23] years); this period was prolonged among adult-onset patients compared with infantile- and juvenile-onset groups (Table 1). Qualitative description of first event, NP-C diagnosis, miglustat treatment initiation, last contact, and death for individual patients is presented in Fig. 1. Overall, 13 patients died during the study period. Causes of death included respiratory infection (*n* = 6), aspiration pneumonia (*n* = 2), pneumonia and entering a vegetative state (*n* = 2), septicemia (*n* = 2), and failure of acid-base homeostasis (*n* = 1).

### Signs and symptoms

**Fig. 2:**
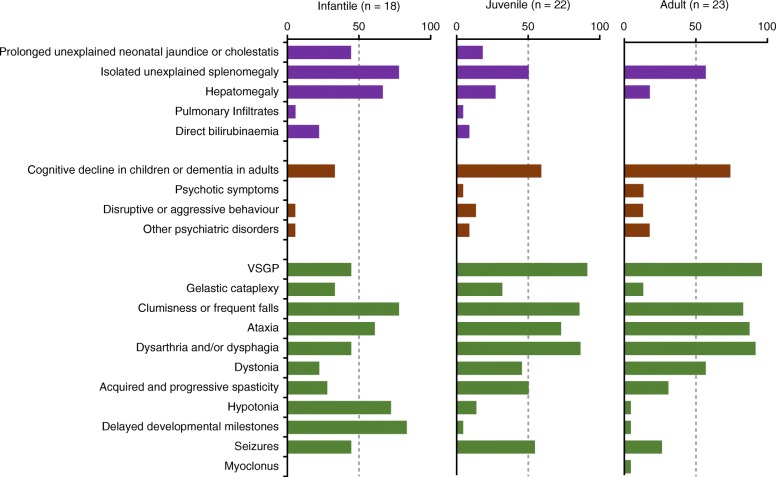
Proportion of patients with visceral, psychiatric and neurologic signs and symptoms by age group. VSGP, vertical supranuclear gaze palsy

The time between onset of symptoms and diagnosis varied across the age groups. In the infantile group, the mean (SD) age of diagnosis was 2.15 (1.10) years and the most common (≥ 50%) visceral symptoms were splenomegaly and hepatomegaly, whereas the most common neurologic symptoms were clumsiness/frequent falls, ataxia, hypotonia, and delayed developmental milestones (Fig. 2). Patients in the juvenile group were diagnosed at a mean (SD) age of 10.97 (3.82) years; the most common neurologic symptoms were VSGP, clumsiness/frequent falls, ataxia, dysarthria/dysphagia, and seizures. Visceral symptoms were still apparent in many patients, and psychiatric symptoms (cognitive decline) were more common than in infantile patients (Fig. 2). Adult patients were diagnosed at a mean (SD) age of 25.98 (8.15) years, and presented with similar symptoms as observed in the juvenile group, but with a higher frequency of psychotic symptoms (Fig. 2).

### Onset of symptoms relative to diagnosis

**Fig. 3:**
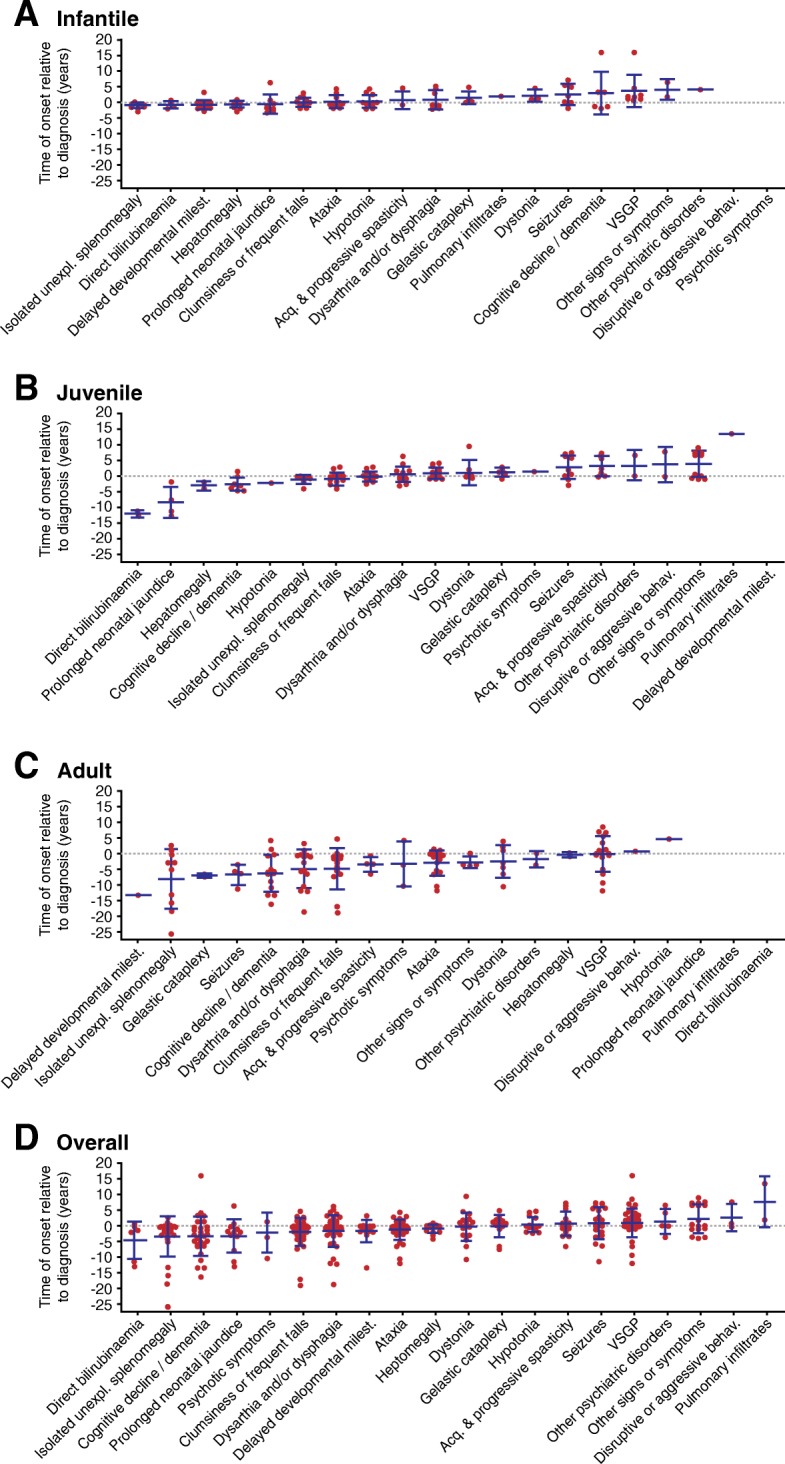
Onset of visceral, psychiatric and neurologic symptoms relative to point of diagnosis in (**a**) Infantile, (**b**) Juvenile, (**c**) Adult and (D) Overall patient groups. VSGP, vertical supranuclear gaze palsy

In infantile patients, visceral signs that include hepatosplenomegaly, direct bilirubinemia, and jaundice typically preceded diagnosis, as well as delayed developmental milestones. However, other neurologic signs such as ataxia, clumsiness/frequent falls, or spasticity were typically noted around or shortly after the time of diagnosis (Fig. 3a and Additional file 1: Table S3). The pattern of presenting symptoms was similar in juvenile patients, albeit with a longer period between onset of visceral symptoms and eventual diagnosis. Neurologic signs were again noted at, or shortly after, the date of diagnosis, in addition to psychiatric manifestations in some patients (Fig. 3b and Additional file 1: Table S3). Patients with NP-C diagnosed in the adult period typically presented with a variety of visceral, neurologic, and psychiatric signs in the years/decades preceding diagnosis (Fig. 3c and Additional file 1: Table S3).

### Disease progression and mortality

**Fig. 4:**
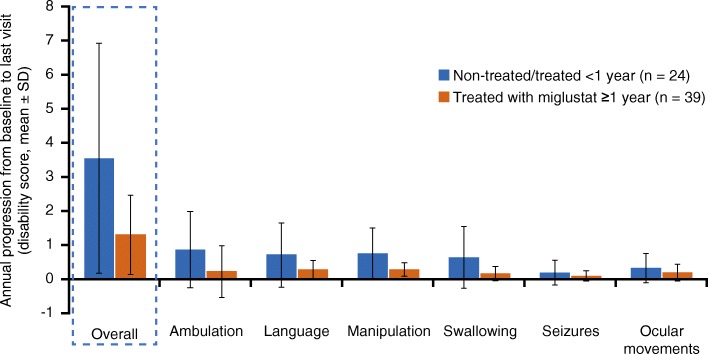
Annual disease progression in miglustat-treated patients vs those non-treated or treated < 1 year. SD, standard deviation

Mean (SD) annual disease progression from baseline to last visit was higher in control patients (3.54 [3.36] points/year) than in treated patients (1.32 [1.15] points/year), though this difference was not statistically significant (Fig. 4). The domains that showed the largest difference in mean progression (disability scale points/year) between treated and control patients were ambulation (0.64), swallowing (0.47), manipulation (0.46), and language (0.43). The progression of ocular movements (0.13) and seizures (0.09) was less affected by miglustat treatment.

**Fig. 5:**
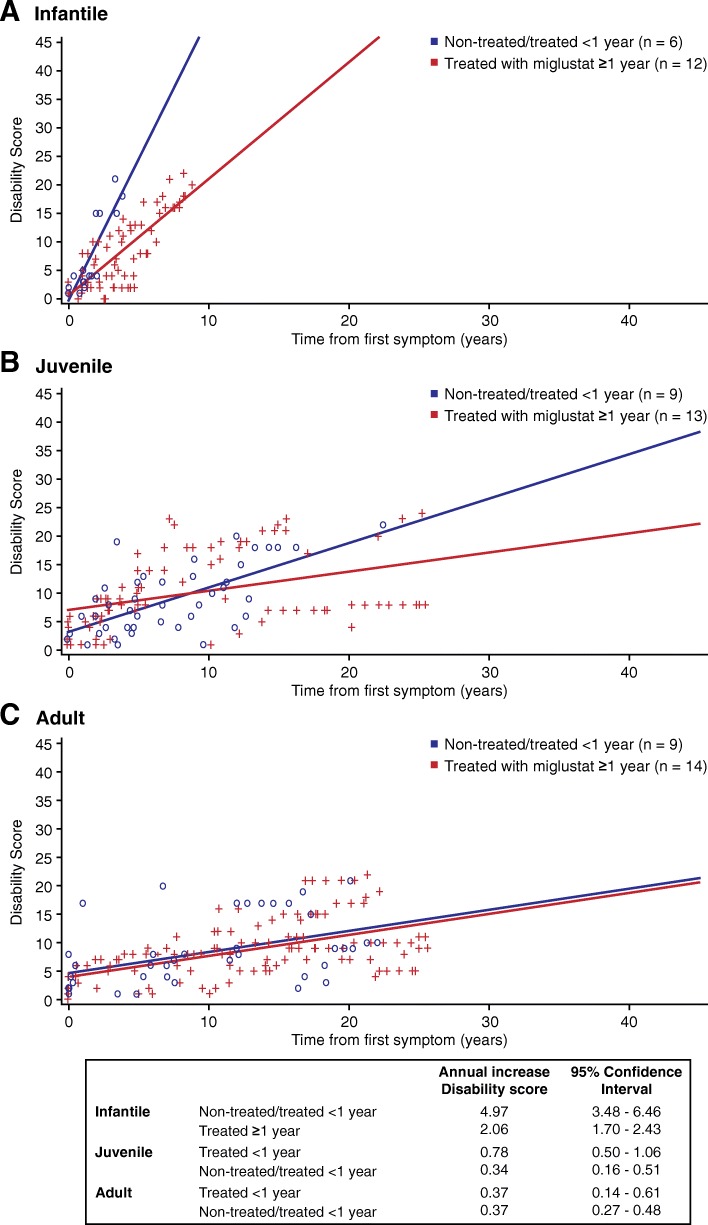
Linear regression analysis showing disease progression pattern in (**a**) Infantile, (**b**) Juvenile and (**c**) Adult age groups

Linear regression analysis was used to further assess miglustat treatment effect on disease progression (Fig. 5). Disease progression was significantly (*p* < 0.001) reduced with miglustat treatment in infantile patients compared with the age-matched control group (increase in annual disability score [95% CI]: 2.06 [1.70–2.43] and 4.97 [3.48–6.46], respectively; Fig. 5). In juvenile patients, a clear trend towards reduced disease progression with miglustat treatment compared with the control group was observed, although this was not significant (increase in annual disability score [95% CI]: 0.34 [0.16–0.51] and 0.78 [0.50–1.06], respectively; Fig. 5). In the adult group, the difference with miglustat treatment compared with control was not significant (increase in annual disability score [95% CI]: 0.371 [0.27–0.48] and 0.374 [0.14–0.61], respectively).

**Fig. 6:**
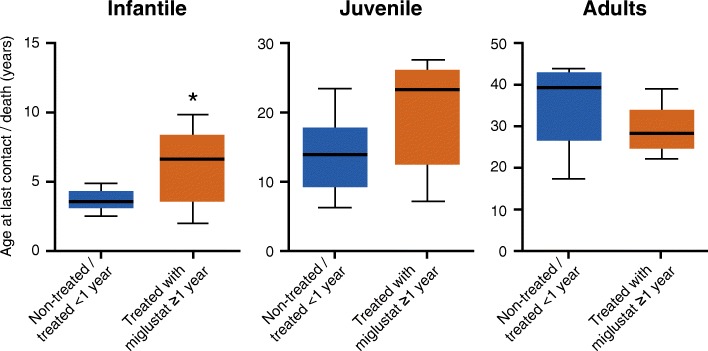
Age at last contact or death showing median survival at 25, 50, and 75% mortality**.** *p = 0.0313. *p* = 0.0735 for juvenile group and *p* = 0.0887 for adult group**.** Box whiskers showing median with 25–75 percentile and max-min

Population survival by age at last contact or death showed a reduction/delay in mortality in treated infantile patients (*p* = 0.031) and a trend towards reduction/delay in mortality in juvenile (*p* = 0.073) patients relative to the control group (Fig. 6).

## Discussion

This retrospective chart review explored the characteristic symptomology of patients with NP-C in different age groups. Overall, the type and onset of symptoms varied across the age groups and are in broad agreement with descriptions of NP-C within the literature [3, 9]. Symptoms that typically precede diagnosis include visceral symptoms in infantile patients, visceral and neurologic signs in juvenile patients, and a varying degree of visceral, psychiatric, and neurologic signs in adult patients. In the current study, patient categorization by age group was based on the date of diagnosis rather than the first symptom occurrence, because for many patients, first symptoms are generally mild and/or non-specific and may only be attributed to NP-C in hindsight upon diagnosis. The 4-year cut-off used in this study differed from the classical early and late infantile age groups, and was selected due to the shift in typical symptomology of NP-C that occurs at around 4 years of age [8, 9]. The pattern of onset of symptoms observed in this study is largely in agreement with the literature, where it is reported that patients who develop NP-C during early infancy often present with predominantly visceral manifestations followed by neurologic signs, whereas adolescent and adult patients present with varying combinations of progressive neurologic signs and psychiatric disturbances [3, 8, 19].

As NP-C is a rare disease, patient population sizes are limited and recruiting sufficient patient numbers for a prospective study is very challenging. This real-world, multicenter chart review of patient data from expert centers allowed a more detailed analysis of miglustat treatment and factors that affected disease progression and survival. Although data were pooled, two additional secondary outcomes that aimed to identify characteristics and factors related to survival (including the factors that differ between control and treated patients) and a retrospective comparison of miglustat treatment with pre-miglustat treatment, could not be assessed due to low numbers of patients with evaluable data. One notable limitation is the inexact agreement between the 3 NP-C disability scales that were used to map clinical data onto the unified NP-C disability scale. Data were obtained from medical records, and were imputed and mapped to obtain additional disability scale assessments. Also, there was a lack of complete medical history for older records. Nevertheless, it should be noted that the reduced rate of disease progression (Fig. 4) correlated with a higher age at last contact or death (Fig. 6), thus validating the use of disability scales to monitor disease evolution or worsening over time, and potentially as tools to predict life expectancy.

In the control group, approximately 20% of patients were prescribed miglustat, but these patients were considered non-treated (treated < 1 year) based on the assumption that the effects of miglustat treatment are not apparent for at least 12 months after treatment initiation [7, 18]. Disability scores from baseline deteriorated more slowly in patients who received miglustat for ≥1 year than in control patients who were not treated or treated < 1 year (Fig. 4). This finding was considered clinically significant but did not achieve statistical significance due to the large patient-to-patient variability and the rather low number of patients, particularly in the control group versus the treated group (*n* = 24 vs *n* = 39, respectively).

In patients treated for ≥1 year, linear regression modeling showed that miglustat treatment reduced disease progression in infantile patients and showed a trend towards reduced disease progression in juvenile patients in comparison with those who had no treatment or were treated for < 1 year. This trend was not seen in the adult group, possibly because the late onset of symptoms may lead to diagnosis when the disease is already at an advanced stage and therefore less amenable to treatment. Furthermore, patients in the infantile and juvenile groups are likely to follow a more severe and rapidly progressive disease course; in these patients the effects of treatment on disease progression are likely to be more pronounced than in adult-onset disease, which progresses more slowly.

## Conclusions

Pooled data from five patient cohorts provide further clarification on the natural history of NP-C and are in agreement with the published literature. These data confirm that, in infantile and juvenile patients, visceral signs often predate diagnosis, whereas neurologic signs typically occur at the time of diagnosis; in adult patients, diagnosis is predated by a wide range of predominantly neurologic and psychiatric signs. Miglustat treatment is associated with a significant reduction in the rate of disability score worsening in infantile patients, and a trend towards reduced disability score worsening in juvenile patients. Both findings are in good agreement with an increased age at last contact or death, thereby supporting the use of miglustat for the treatment of NP-C.

## Additional file

Additional file 1: **Table S1.** Mapping of existing disability scales to the unified NP-C disability scale. **Table S2.** Medical history events computed into the 6-domain disability scale. **Table S3.** Mean onset of visceral, psychiatric and neurologic symptoms relative to point of diagnosis. (DOCX 33 kb)

## Abbreviations

CI: Confidence interval
EDC: Electronic data capture
NP-C: Niemann-Pick disease Type C
SD: Standard deviation
VSGP: Vertical supranuclear gaze palsy
