# Supplementary material for: Disease characteristics, prognosis and miglustat treatment effects on disease progression in patients with Niemann-Pick disease Type C: an international, multicenter, retrospective chart review
Source: Orphanet J Rare Dis. 2019 Feb 7;14:32. doi: 10.1186/s13023-019-0996-6 (PMC6367842; doi:10.1186/s13023-019-0996-6)
Supplement: Supplementary file 1 — Table S1. Mapping of existing disability scales to the unified NP-C disability scale. Table S2. Medical history events computed into the 6-domain disability scale. Table S3. Mean onset of visceral, psychiatric and neurologic symptoms relative to point of diagnosis. (DOCX 33 kb) [file 13023_2019_996_MOESM1_ESM.docx]

**Additional file 1: Table S1** Mapping of existing disability scales to the unified NP-C disability scale

| **Disability score item** | **Severity** | **Unified disability scale** | **Iturriaga scale (15)** | **Pineda scale (14)** | **Fecarotta scale (17)** | **Notes** |
| --- | --- | --- | --- | --- | --- | --- |
| Ambulation | Normal, absence of abnormalities | **0** | 1 | *n/a* | 0 |  |
|  | Clumsiness | **1** | *–* | 1 |  |  |
|  | Autonomous ataxic gait | **2** | 2 | 2 | 1 |  |
|  | Outdoor assisted ambulation | **3** | 3 | 3 |  |  |
|  | Indoor assisted ambulation | **4** | 4 | 4 | *2* | *Deviating definition by Fecarotta: "assisted ambulation"* |
|  | Wheelchair bound | **5** | 5 | 5 | 3 |  |
| Manipulation | Normal, absence of abnormalities | **0** | 1 | *n/a* | 0 |  |
|  | Tremor | **1** | *–* | 1 |  |  |
|  | Slight dysmetria / dystonia (allows autonomous manipulation) | **2** | 2 | 2 | 1 | *Deviating definition by Fecarotta: "mild difficulties in reaching the nose or an object"* |
|  | Mild dysmetria / dystonia (req. help for several tasks but able to feed himself) | **3** | 3 | 3 | 2 | *Deviating definition by Fecarotta: "moderate difficulties in reaching the nose or an object"* |
|  | Severe dysmetria / dystonia (req. assistance in all activities) | **4** | 4 | 4 | 3 | *Deviating definition by Fecarotta: "severe dysmetria, preventing the patient from reaching nose or an object"* |
| Dystonia | Normal, absence of abnormalities | *–* | *–* | *–* | 0 | *Not separately covered by Iturriaga and Pineda* |
|  | Slight dystonia, not disabling, interfering with activities | *–* | *–* | *–* | 1 |  |
|  | Dystonia on action of distant part of body or intermittently at rest, interfering with normal activities and causing moderate impairment of function | *–* | *–* | *–* | 2 |  |
|  | Severe dystonic movements present at rest, complete impairment of function | *–* | *–* | *–* | 3 |  |
| Language | Normal, absence of abnormalities | **0** | 1 | *n/a* | 0 |  |
|  | Delayed acquisitions | **1** | *–* | 1 |  |  |
|  | Mild dysarthria (understandable) | **2** | 2 | 2 | 1 |  |
|  | Severe dysarthria, poor comprehensive language | **3** | 3 | 3 | 2 |  |
|  | Non-verbal communication, anarthria | **4** | 4 | 4 | 3 |  |
|  | Absence of communication | **5** | 5 | 5 |  |  |
| Swallowing | Normal, absence of abnormalities | **0** | 1 | *n/a* | 0 |  |
|  | Difficulty in chewing (abnormal), long feeding times | **1** | *–* | 1 | 1 |  |
|  | Occasional dysphagia | **2** | 2 | 2 | *2* | *Deviating definition by Fecarotta: "arching or stiffening of the neck during feeding, gurgly, hoarse, or breathy voice quality"* |
|  | Daily dysphagia | **3** | 3 | 3 | *3* | *Deviating definition by Fecarotta: "excessive drooling or food/liquid coming out of the mouth or nose, coughing or gagging during meals, difficulty breastfeeding"* |
|  | Unable to swallow, nasogastric tube or gastric button feeding | **4** | 4 | 4 | 4 |  |
| Seizures | Normal, absence of abnormalities | **0** | *–* | *n/a* | *–* | *Not covered by Iturriaga and Fecarotta* |
|  | Occasional seizures | **1** | *–* | 1 | *–* |  |
|  | Seizures with antiepileptic drugs | **2** | *–* | 2 | *–* |  |
|  | Seizures resistant to antiepileptic drugs | **3** | *–* | 3 | *–* |  |
| Ocular movements | Normal, absence of abnormalities | **0** | *–* | *n/a* | *–* | *Not covered by Iturriaga and Fecarotta* |
|  | Slow ocular pursuit | **1** | *–* | 1 | *–* |  |
|  | Vertical ophthalmoplegia | **2** | *–* | 2 | *–* |  |
|  | Complete ophthalmoplegia | **3** | *–* | 3 | *–* |  |
| Developmental delay / cognitive impairment | Normal, absence of abnormalities | *–* | *–* | *–* | 0 | *Not covered by Iturriaga and Pineda* |
|  | Mild cognitive impairment/psychomotor delay | *–* | *–* | *–* | 1 |  |
|  | Moderate cognitive impairment/psychomotor delay | *–* | *–* | *–* | 2 |  |
|  | Severe cognitive impairment/psychomotor delay | *–* | *–* | *–* | 3 |  |

n/a, not applicable; NP-C, Niemann-Pick disease Type C; *–*, severity description not included in disability scale

**Additional file 1: Table S2** Medical history events computed into the 6-domain disability scale

| **Symptom in medical history** | **Disability scale domain** | **Disability scale score** |
| --- | --- | --- |
| Prolonged unexplained neonatal jaundice or cholestasis |  |  |
| Isolated unexplained splenomegaly |  |  |
| Hepatomegaly |  |  |
| Pulmonary infiltrates |  |  |
| Direct bilirubinemia |  |  |
| Vertical supranuclear gaze palsy | Ocular Movements | **2** |
| Gelastic cataplexy |  |  |
| Clumsiness or frequent falls | Ambulation | **1** |
| Ataxia | Ambulation | **2** |
| Dysarthria and/or dysphagia | Language / Swallowing | **2/2** |
| Dystonia | Manipulation | **2** |
| Acquired and progressive spasticity |  |  |
| Hypotonia |  |  |
| Delayed developmental milestones | Language | **1** |
| Seizures | Seizures/Epilepsy  + antiepileptic drugs  + multiple? antiepileptic or drug increase | **1 2 3** |
| Myoclonus | Seizures | **1** |
| Cognitive decline in children or dementia in adults |  |  |
| Psychotic symptoms |  |  |
| Treatment-resistant psychiatric symptoms |  |  |
| Disruptive or aggressive behavior |  |  |
| Progressive psychiatric disorders |  |  |
| Other psychiatric disorders |  |  |
| Other symptoms: Epilepsy | Seizures/Epilepsy  + antiepileptic drugs  + multiple? antiepileptic or drug increase | **1 2 3** |
| Other symptoms: very severe  dystonic form | Manipulation | **4** |

**Additional file 1: Table S3** Mean onset of visceral, psychiatric and neurologic symptoms relative to point of diagnosis

| **Symptoms** | **Infantile** | **Juvenile** | **Adult** | **Overall** |
| --- | --- | --- | --- | --- |
|  | **Mean (SD), years** | | | |
| Prolonged neonatal jaundice | −0.48 (3.08) | −8.47 (4.86) | NR | −3.14 (5.29**)** |
| Isolated unexplained splenomegaly | −0.84 (0.88) | −1.18 (1.44) | −8.02 (9.55) | −3.31 (6.37) |
| Hepatomegaly | −0.55 (1.06) | −3.01 (1.54) | −0.29 (0.82) | −0.83 (1.33) |
| Pulmonary infiltrates | 2.00 (0.00) | 13.49 (0.00) | NR | 7.74 (8.12) |
| Direct bilirubinemia | −0.69 (1.20) | −12.09 (1.11) | NR | −4.49 (5.98) |
| VSGP | 3.73 (5.12) | 0.85 (1.73) | −0.06 (5.65) | 1.04 (4.57) |
| Gelastic cataplexy | 1.52 (2.00) | 1.18 (1.43) | −6.92 (0.64) | −0.03 (3.55) |
| Clumsiness or frequent falls | 0.10 (1.43) | −0.95 (1.98) | −4.78 (6.60) | −1.85 (4.45) |
| Ataxia | 0.35 (2.05) | −0.21 (1.60) | −2.96 (4.15) | −1.15 (3.26) |
| Dysarthria and/or dysphagia | 0.95 (3.08) | 0.52 (2.45) | −4.83 (6.08) | −1.57 (5.08) |
| Dystonia | 2.27 (2.00) | 1.04 (3.98) | −2.44 (5.19) | −0.18 (4.54) |
| Acquired & progressive spasticity | 0.74 (2.84) | 3.17 (3.18) | −3.42 (2.36) | 0.76 (3.89) |
| Hypotonia | 0.41 (2.01) | −2.25 (0.00) | 4.73 (0.00) | 0.52 (2.32) |
| Delayed developmental milestone | −0.67 (1.47) | NR | −13.26 (0.00) | −1.51 (3.55) |
| Seizures | 2.61 (3.37) | 2.75 (3.69) | −6.68 (3.28) | 0.91 (5.04) |
| Cognitive decline / dementia | 3.05 (6.80) | −2.63 (2.03) | −6.23 (5.95) | −3.19 (6.24) |
| Psychotic symptoms | NR | 1.40 (0.00) | −3.22 (7.25) | −2.07 (6.36) |
| Disruptive or aggressive behavior | NR | 3.70 (5.65) | 0.82 (0.00) | 2.74 (4.33) |
| Other psychiatric disorders | 4.20 (0.00) | 3.31 (4.68) | −1.73 (2.63) | 1.47 (3.98) |
| Other signs or symptoms | 4.19 (3.29) | 3.85 (4.19) | −2.71 (1.90) | 2.35 (4.55) |
| NR, not reported; SD, standard deviation; VSGP, vertical supranuclear gaze palsy | | | | |
